# The Effect of Correlated Colour Temperature on Physiological, Emotional and Subjective Satisfaction in the Hygiene Area of a Space Station

**DOI:** 10.3390/ijerph19159090

**Published:** 2022-07-27

**Authors:** Ao Jiang, Xiang Yao, Stephen Westland, Caroline Hemingray, Bernard Foing, Jing Lin

**Affiliations:** 1 International Lunar Exploration Working Group, EuroMoonMars at The European Space Research and Technology Centre, European Space Agency, 2200 AG Noordwijk, The Netherlands; foing@strw.leidenuniv.nl; 2 School of Design, University of Leeds, Leeds LS2 9JT, UK; s.westland@leeds.ac.uk (S.W.); c.s.hemingray@leeds.ac.uk (C.H.); lj282101518@gmail.com (J.L.); 3 School of Mechanical Engineering and Mechanics, Xiangtan University, Xiangtan 411105, China; 4 Faculty of Science, Leiden University, 2311 EZ Leiden, The Netherlands; 5 Faculty of Science, Vrije Universiteit Amsterdam, 1081 HV Amsterdam, The Netherlands

**Keywords:** correlated colour temperature, physiological responses, emotion, space station hygiene area

## Abstract

The hygiene area is one of the most important facilities in a space station. If its environmental lighting is appropriately designed, it can significantly reduce the psychological pressure on astronauts. This study investigates the effect of correlated colour temperature (CCT) on heart rate, galvanic skin response, emotion and satisfaction in the hygiene area of a space station. Forty subjects participated in experiments in a hygiene area simulator with a controlled lighting environment. The lighting conditions included 2700 K, 3300 K, 3600 K, 5000 K and 6300 K; physiological responses (heart rate, galvanic skin response), as well as emotion and satisfaction, were recorded. The results showed that CCT significantly influenced the participants’ physiological and subjective responses in the space station hygiene area. 6300 K led to the best emotion and satisfaction levels, the highest galvanic skin response and the lowest heart rate. The opposite was true for 2700 K.

## 1. Introduction

In long-term manned space missions, astronauts may suffer unstable physiological and psychological states in confined, narrow, isolated environments, which brings unprecedented challenges to space mission support and manned space engineering [1,2,3]. As an important measure for ensuring the success of manned space missions, habitability research needs to consider the harmony of the astronauts’ body mechanics, but also their psychological and physical health [4,5]. Many NASA reports indicate that habitability is highly related to human factors, behavioural health, environmental interaction and human-computer interaction [6]. The goal of habitability is to provide astronauts with physical, psychological, social and spiritual support. As early as 1985, NASA’s habitability requirements for long-term manned spacecraft included not only operational requirements, but also requirements related to long-term sustainable living [7,8,9]. Besides, for spacecraft designers, the space, layout, lighting, colour, air circulation and smell of the environment have an important impact on the level of habitability. Environmental factors such as lighting and colour directly affect human visual functions and are listed as the focus of habitability [10,11]. Therefore, lighting, and particularly the correlated colour temperature (CCT) of lighting, can play a significant role in emotional regulation [6,11] and potentially affects the habitability level of a space station [12]. A number of studies have been conducted to quantify the relationship between different CCT lighting conditions in space stations and their effects on human psychological and physiological responses [13,14].

Light has been shown to have strong non-visual effects on a range of biological functions, such as the regulation of human emotion and the circadian system [15]. CCT is a key factor of light and has been shown to affect human physiology as well as psychology [16]. In manned spacecraft, most lighting uses fluorescent or LED technology. Currently, the International Space Station (ISS) uses solid-state lighting assemblies (SSLA) equipped with solid-state light emitting diodes (LEDs) to illuminate the astronauts’ working and living environments [17]. This new lighting system offers a wide range of colour temperature adjustments that can aid astronauts’ vision and may be used as a lighting countermeasure against the emotional stress and performance degradation caused by isolation, confinement and lack of social support on board the ISS [18]. Several studies have tested SSLA in space and found that the system could be used to aid astronauts’ vision and as an in-flight countermeasure to circadian rhythm disruption, sleep disruption and cognitive performance deficits on the ISS, thereby improving the habitability of the ISS [19].

In ground environments, some literature suggests that colour temperature has an important effect on human cognitive abilities and emotion. Higher colour temperatures (≥4000 K) approximate blue-white light and are often referred to as cool colours, while lower colour temperatures (≤3000 K) approximate red light and are referred to as warm colours [20,21]. Several studies have shown that warm colours (3000 K) significantly induce negative emotions when humans are engaged in cognitive tasks, compared to cool fluorescent lights (4000 K) [21,22]. Besides, two studies comparing the effects of fluorescent and LED light sources of different colour temperatures showed that LED light sources significantly reduced participants’ fatigue and enhanced cognitive performance, especially at higher colour temperatures [23,24]. Furthermore, other studies have found an effect of colour temperature on physiological performance and subjective emotion [21,25,26]. Extensive studies have found that CCT affects human thermal comfort, particularly in enclosures similar to spacecraft, and some studies have shown that people feel warmer and more comfortable under yellow light than under blue light. This effect of CCT is small but significant [6].

Comfortable CCT can increase satisfaction and happiness. Veitch (2001) found that office lighting conditions affect positive mood and happiness [27]. People working in offices with higher-quality lighting found the space more attractive, were in a happier mood and showed a significant sense of well-being. In addition to subjective emotional responses, a range of physiological responses have also been associated with the CCT characteristics of lighting. These include, e.g., core body temperature, heart rate, brain waves, galvanic skin response (GSR) [28,29,30], rectal temperature [31] and melatonin suppression. Previous studies have demonstrated that short-wavelength light has a greater effect on core body temperature and heart rate than long-wavelength light [32]. The reason for this phenomenon is that CCT can influence hypothalamic activity, which regulates and controls body temperature through the secretion of melatonin [33].

Some studies have suggested that the CCT of lighting in a ground environment has a significant impact on how people feel when they use bathrooms or toilets, with lower colour temperatures of around 3000 K usually giving a greater sense of comfort and people preferring warm colours in bathroom lighting environments. As a result, some hotels or luxury flats have warm colour lighting in bathrooms to provide a good visual perception and experience for their guests [34,35,36]. However, the characteristics of the space station environment are quite different from those of ground environments, as its primary objective is to ensure crew safety and operational reliability in a confined, isolated and extreme environment. A separate but essential element of the crew’s daily life is the space station’s hygiene area, which, in addition to the normal collection of crew excrement and waste, also recycles the crew’s urine to generate drinking water. Although the literature is sparse, what is known and what has recently been reported by crew members of the International Space Station (ISS) and the Chinese Space Station suggests that the small, enclosed environment of the hygiene area has a negative impact on the crew’s ability to operate and defecate. Secondly, the environment has many stressors, such as noise from the urination and defecation units, unpleasant odours and the microgravity environment, which can cause operational difficulties. In addition, due to the complexity of the equipment in the hygiene area environment, crew members need to follow strict equipment guidelines when performing defecation, which results in the basic defecation process or wash-up process often taking longer to complete than on the ground. As a result, the hygiene area’s lighting environment is likely to affect their physiological needs, information acquisition and judgement, and emotional experience [37,38,39,40]. Based on previous findings, we expected CCT to have an impact on physiological responses and mood. Therefore, the aim of this study was to explore the effects of five different CCTs on participants’ heart rate (HR), galvanic skin response (GSR), emotion and satisfaction while using a urine/stool collector in a simulated space station hygiene area.

## 2. Materials and Methods

### 2.1. Participants

A total of 40 healthy Chinese participants (12 female and 28 male) aged 33–49 years (36.5 ± 2.5 y (mean ± SD)) took part in the study, as this is the age group to which the majority of current reserve astronauts and crew members belong. All participants were non-smokers and free from medical, psychiatric and sleep disorders, as assessed by an interview and two screening questionnaires, including the Pittsburgh Sleep Quality Index and the Horne–Ostberg Morning–Eveningness Questionnaire [41,42]. The participants had normal or corrected-to-normal visual acuity better than 0.6 (1/minute of visual angle) [43,44] and normal colour vision. as assessed by the Ishihara plate test (international 36-plate version) before and after the study. They were trained to use the CAPTIV human factors data acquisition system (CAPTIV-L7000, France) and the urine/stool collector simulator (self-developed). During the entire simulation experiment task, the participants were asked to refrain from caffeine and alcohol intake for 24 h prior to the experiment and maintain a regular work–rest schedule and a normal diet and water intake. They went to bed at approximately 10 p.m. and woke up at 6 a.m. [45]. An information sheet was provided, and the participants first read and signed an informed consent form providing information about the study and their rights as participants in accordance with the guidelines of the Helsinki Declaration. The study was approved by the University of Leeds Ethics Committee.

### 2.2. Experimental Set-Up and Scene

**Figure 1:**
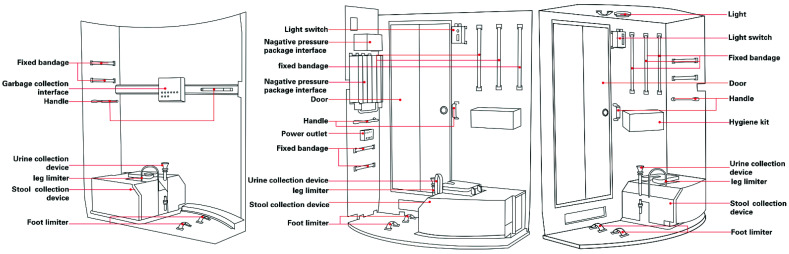
Exterior view of the hygiene area and drawings of the simulator.

A controlled experimental environment was designed and built based on the hygiene area of a space station with support from the China Astronauts Centre (Figure 1). Based on the reports on the International Space Station by Messerschmid and Borrego [4,46,47], the various functional elements were optimised to provide an integrated experimental system of the hygiene area in the space station in order to ensure reliability and accuracy of the experiment. This simulated environment included four subsystems: temperature and humidity monitoring, illumination system, physiological monitoring, and urine/stool collector simulator.

**Figure 2:**
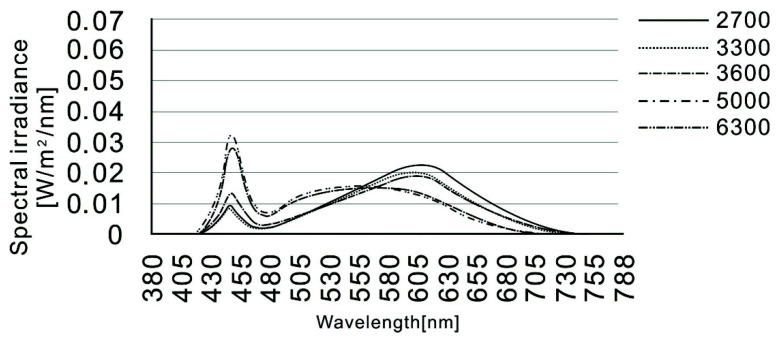
Spectral distribution of the light in the test room at nominal CCTs of 2700 K, 3000 K, 3600 K, 5000 K and 6300 K.

The experiments were carried out using a simulated lighting system (LED, supplied by Touslite Lighting System, TLS Lighting, Badersfield, UK) for the hygiene area. All equipment was new and installed one month prior to the official experiment. Table 1 shows the Duv and illuminance for the five lighting conditions. The spectral distributions of the LED lighting at the five different colour temperatures are shown in Figure 2. The illuminance was measured at eye level with a calibrated spectroradiometer (JETI Specbos 1201, JETI Technische Instrumente GmbH, Jena, Germany) and the luminaire was switched on for 30 min before each session to stabilise the illuminance. The walls and ceiling were off-white with 72% and 67% reflectance, respectively. The light grey floor had a reflectance of 31% and the off-white operating panel had a reflectance of 66%. The luminance images were captured using a TechnoTeam LMK Mobile (TechnoTeam, Ilmenau, Germany) prior to the experiments, which captured a fairly even distribution of luminance at the floor and wall locations, between approximately 250–300 cd/m^2^ on the floor and between 125 and 250 cd/m^2^ on the walls. The lighting system was mounted on the ceiling and was clearly visible to the participants, although their main field of vision was the operating panel in front of them.

Temperature and humidity were controlled using a closed-loop method. The temperature and humidity data was collected in real time using temperature sensors (Yundian Hi-Tech Sensor, DFN-3l, China) and humidity sensors (HTU20D, France). When the temperature and humidity data collected differed from the target temperature (19~26 °C) and humidity (30~70%) [4], the temperature and humidity control centre activated a HULL hot air blower (wth-fh13, USA) and a GREE dehumidifier (dh12en, China). Noise in the hygiene area came mainly from noise (42–45 db) emitted by the urinal collector during operation [4].

### 2.3. Experimental Procedure

**Figure 3:**
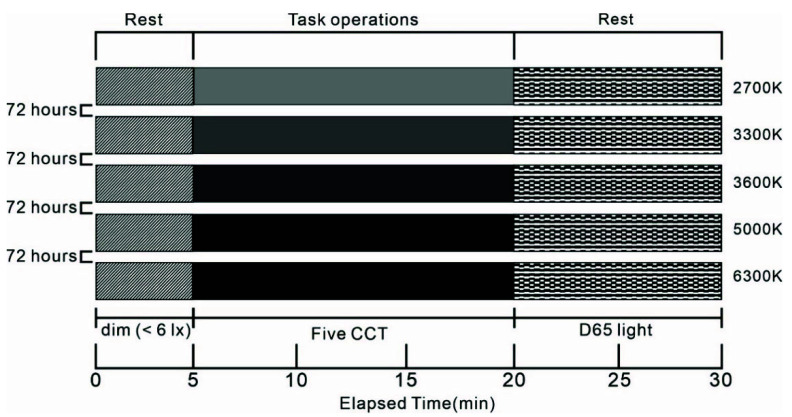
Experimental designs.

**Figure 4:**
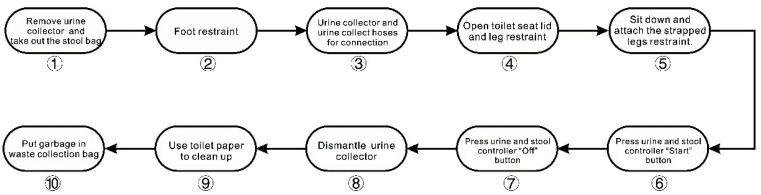
Hygiene area operating procedures.

(1) The day before the experiment, the participants were asked to get enough rest. They were to keep calm and familiarise themselves with the test environment and the operation process of the urine/stool collector simulator before the experiment in order to avoid changes in their psychological state in an unfamiliar environment.

(2) We used a randomised within-subject study design. The study consisted of five sessions, separated by at least 72 h for each participant, to avoid potential carry-over effects (Figure 3). The participants were admitted to the laboratory at noon (approximately 3.5–5 h after waking up). The laboratory had been set up to simulate the external environment of the hygiene area of the space station to ensure that the participants had an appropriate environmental experience before they entered the simulated hygiene area. After connecting the T-Sens RF sensor, the HR sensor and the GSR sensor to each participant, the participants were exposed to dim (<6 lx) polychromatic white light for 5 min to relieve emotions [48,49]. After the adaptation period, the HR and GSR of the 40 participants in the five CCT lighting environments were recorded for about 15 min. The 15-min experiment length was chosen because, typically, the average time spent by astronauts while performing hygiene area manipulation behaviours is around 15 min. Had the length of the experiments been too long or too short, it would not have reflected the crew’s experience in that environment well. There are, however, some CCT lighting studies that arrived at significant results with shorter experiment lengths [50,51]. During the experiments, the participants strictly followed the processes for operating and using the urine/stool collector; the specific operation steps are shown in Figure 4. This was followed by the completion of the PANAS and CCTSV questionnaires. For each participant, the order of the CCT conditions was randomised to avoid potential sequence effects. At the end of the test, the participants rested in a D65 light environment for ten minutes and then left the laboratory.

### 2.4. Materials and Data Collection

The data collection combined the physiological measurements. The data monitoring and acquisition system used the CAPTIV human factor data acquisition system (CAPTIV-L7000, France). The following T-Sens sensors were used as wireless data acquisition sensors to measure HR and GSR:

- A T-Sens HR sensor was located on a chest strap;
- The electrode of the T-Sens GSR sensor was connected to the participant’s right hand: on the inside distal phalanges of the index and ring fingers, the skin surface was degreased with medical alcohol and coated with conductive paste to increase the conductive effect.

T-Log was used as a wireless data recorder to feed the collected physiological signals back through the visual interface in real time and store the physiological signals received. The experimental data was transmitted to the computer and processed by the CAPTIV-L2100 software. The HR and GSR sampling frequency was 16 and 32 Hz, respectively. Forty-two CAPTIV-L7000 T-Sens sensors were worn on the corresponding part of the participants’ upper body. The CAPTIV-L7000 software was used to process the data obtained by the T-Sens sensors and the T-Log logger. The CAPTIV-L7000 monitor chest strap can lose direct connection with a participant’s skin. This did happen a few times and caused partial data loss, so the non-usable data was removed before the data analysis.

The PANAS questionnaire has good validity and reliability for measuring subjective emotions [49]. The measurement consists of two dimensions, positive and negative emotions, each measured with nine emotion adjectives. The internal consistency coefficients for the PANAS in this study were α(PA) = 0.90 and α(NA) = 0.92. At the end of each session of the experiment, the participants were asked to assess their emotional state for the session on a 5-point response scale ranging from 1 = not at all to 5 = very much.

The Correlated Colour Temperature Satisfaction Vote (CCTSV) uses a five-point scale consisting of very satisfied (+2), satisfied (+1), neutral (0), dissatisfied (−1) and very dissatisfied (−2). The internal consistency of the scale was set at α = 0.91. The scale has been widely used to measure satisfaction with CCT for lighting [52].

Before the experiment, the experimenter gave the participants a detailed introduction to each questionnaire and the participants filled in the questionnaires according to their true feelings.

### 2.5. Statistical Analyses

The Shapiro–Wilk and Leven’s tests were used to assess the normality and homogeneity of variance, respectively, prior to the application of formal statistics to analyse the data. A one-way ANOVA was conducted to examine the effects of different CCTs on emotion, HR, GRS and satisfaction in order to check whether there were significant differences in emotion, HR, GRS and satisfaction across CCTs and to determine their statistical significance, if any; α < 0.05 was considered statistically significant. Tukey HSD post hoc tests were conducted for pairwise comparisons, and Bonferroni correction was used. SPSS (version 24; IBM Corporation; Armonk, NY, USA) was used for all analyses in this study.

## 3. Results

**Figure 5:**
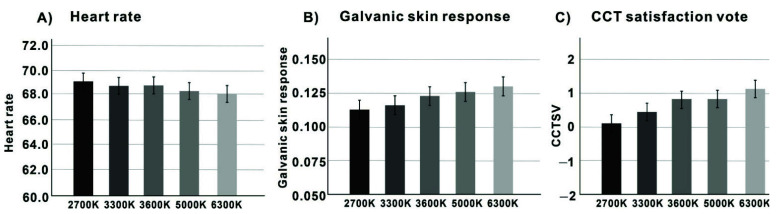
(**A**) Heart rate; (**B**) galvanic skin response; (**C**) CCT satisfaction vote impact in the five CCT conditions (the error bars show the standard error of the means.).

We first analysed the HRs affected by the five CCTs and found that the main effect of CCT on HR was not significant (F = 4.624, *p* = 0.062 > 0.05), indicating that there was no difference in HR between the different CCT conditions. The heart rate for the five CCT conditions is presented in Figure 5A. Further post hoc tests revealed that participants’ HR was lowest at 6300 K compared to the other four CCT lighting conditions but not significantly different (*p* = 0.071 > 0.05) and highest at 2700 K, also not significantly different (*p* = 0.067 > 0.05) compared to the other four CCT lighting conditions. As can be seen from the data, the trend in HR decreased with increasing CCT lighting conditions, except for a slight increase in the 3600 K lighting condition compared to 3300 K.

We analysed the differences in GSR and observed a significant main effect of CCT (F = 5.261, *p* = 0.042 > 0.05), indicating a difference in GSR between the five CCT conditions. The GSR for the five CCT conditions is shown in Figure 5B. Further post hoc analysis showed that participants had a significantly lower GSR in the 6300 K condition compared to the other four CCT conditions (*p* = 0.039 < 0.05), while there was no significant difference between the other four CCT lighting conditions (*p* = 0.051 > 0.05). However, according to the data, the GSR decreased with higher CCT, with the highest GSR (SD: 0.0132, M: 0.002) at 2700 K and the lowest GSR (SD: 0.0114, M: 0.004) at 6300 K.

Based on the HR and GSR data, we found opposite trends in the response of HR and GSR to the different CCT lighting conditions. HR gradually decreased and GSR gradually increased with higher CCT lighting conditions. Furthermore, the effect of the CCT lighting conditions on GSR was more significant than that on HR, and the effect of different CCT lighting conditions on GSR varied considerably. Both physiological signals seem to indicate that CCT lighting conditions do not seem to produce significant arousal affecting HR, whereas they do produce more arousal affecting GSR.

Next, we analysed CCT satisfaction, which also had a significant main effect (F = 3.824, *p* = 0.035 < 0.05), with satisfaction levels for the different CCT conditions shown in Figure 5C. Further post hoc tests revealed that the participants were significantly less satisfied at 2700 K compared to the other four CCT conditions (*p* = 0.031 < 0.05), with no significant difference in satisfaction between the other four CCT conditions (*p* = 0.064 > 0.05), although these rates were numerically highest at 6300 K, which was not significant (*p* = 0.057 > 0.05). Moreover, we found that participants’ satisfaction with the CCT was almost identical when the CCT was at 3600 K and 5000 K. Although the level of satisfaction was slightly lower at 5000 K than at 3600 K, the difference between the two was very slight. This seems to indicate that participants’ satisfaction did not change significantly in the 3600 to 6300 K lighting range.

**Figure 6:**
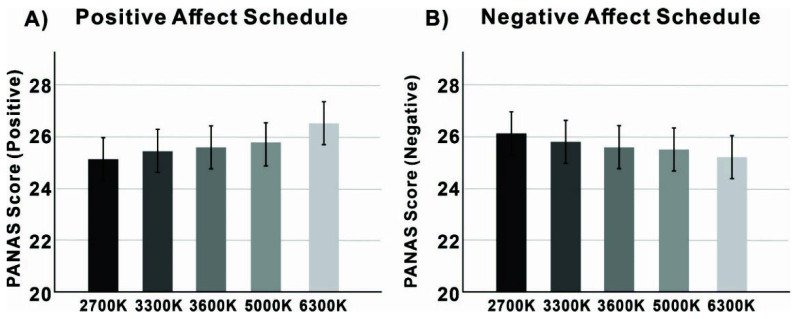
(**A**) Positive emotional impact and (**B**) negative emotional impact in the five CCT conditions (the error bars show the standard error of the means.).

Turning to the affect scales, and starting with positive affect, there was a significant effect of CCT (F = 12.703, *p* = 0.046 < 0.05). The data is illustrated in Figure 6B. Positive affect was significantly higher in the 6300 K condition compared to the other four CCT conditions (*p* = 0.048 < 0.05). There was no significant difference between the other four CCT conditions (*p* = 0.078 > 0.05). It is clear from the data that the higher the CCT, the better the positive emotion, although this was not significant. Besides, we also found a non-significant main effect of CCT on negative emotion (F = 5.147, *p* = 0.083 > 0.05). However, the data showed that the higher the CCT, the lower the negative emotion, with the highest negative emotion at 2700 K and the lowest negative emotion at 6300 K.

## 4. Discussion

To date, little is known about the optimal correlated colour temperature to benefit individual emotion, satisfaction and physiological performance of the crew when operating in the hygiene area of the space station. The aim of this study simulating a space station hygiene area was to explore the effects of lighting under different CCT conditions on the participants’ heart rate, galvanic skin response, emotions and satisfaction levels. The results of the experiment showed that there was a significant difference in GSR, positive emotion and satisfaction under different CCT conditions, in addition to heart rate and negative emotion. GRS, positive emotion and CCT satisfaction all increased with higher CCT conditions. This means that the higher the CCT, the more comfortable and better the participants’ emotions in the space station hygiene area. Furthermore, although HR and negative emotions did not differ significantly across the five CCT conditions, it was clear from the data that HR and negative emotions gradually decreased as CCT increased.

The present study found that the five CCT conditions did not make any significant difference in the participants’ heart rates in the space station hygiene area simulator, although the highest heart rates were found at 2700 K compared to the other CCT conditions and the lowest during 6300 K, although this did not reach statistical significance. These findings are broadly similar to some previously reported results. Some studies found that the heart rate of participants who remained in 6000 K lighting was slightly, but not significantly, higher than during the 2700 K period, regardless of whether it was day or night for the participants [52,53,54]. Conversely, other studies have found an increase in heart rate when CCT lighting conditions were higher [54,55,56], which is not consistent with the results of the present study. This may be due to the variability between CCT lighting conditions, the task scenarios set and the selection of the participant population. However, both the present study and previous studies found that CCT lighting conditions did not significantly affect the heart rate, regardless of whether CCT lighting conditions and heart rate followed the same trend or opposite trends. This is also consistent with the view of some other studies that changes in heart rate are not significantly related to the effects of short-term CCT, suggesting that greater differences in CCT levels or more sustained exposure times may be required to improve performance during the day and achieve more robust results in terms of the heart rate being altered [57]. On the other hand, significant changes in the participants’ GSR were also found in this study, even in short 15-min experiments, with GRS levels increasing with increasing CCT conditions. The GRS level at 6300 K was significantly higher than the GRS level at 2700 K. This replicates and extends the outcomes from previous studies, where Smolders (2017) found in a study of human alertness and cognitive performance that human GRS was significantly lower in low CCT conditions than in high CCT conditions [48]. There are also studies where the 6000 K condition was assessed as being more activating, which confirms the activation results for the GRS [58,59].

Considering that different CCT conditions have an effect on human physiological performance, we further examined the effect of CCT conditions on emotions and satisfaction levels. Our study found that participants’ satisfaction levels and positive emotions while operating in the space station hygiene area increased with increasing CCT levels. In particular, regarding CCT satisfaction levels, the participants had the highest satisfaction level at 6300 K and significantly lower satisfaction levels at 2700 K. They also had the highest positive effect at 6300 K and showed a significant increase in positive affect at 6300 K. This is similar to some previous studies where daytime exposure to higher CCT had beneficial effects on alertness and emotion in humans, and participants felt more alert, energetic and happy compared to lower CCT conditions [60,61,62,63]. However, other studies have suggested that in lighting conditions above 6000 K, participants also experience fewer positive effects and more negative effects, regardless of the time and duration of exposure [52]. This discrepancy can perhaps be gleaned from the reports of the participants in this study. Due to the nature of the space station hygiene area, the participants felt that the area was a small space and an enclosed and isolated environment, making it more complex to operate and more difficult to operate than in a normal bathroom on the ground. They agreed that the high CCT lighting conditions allowed them to operate and respond quickly and effectively with less repetition of operations. This may account for the difference in findings compared to some previous studies. Besides, some of the current literature on the International Space Station (ISS) reports that the operating mode in the new ISS LED system is set at 6500 K to help the crew optimise efficiency, improve alertness and reduce error rates [64,65,66]. This also indirectly validates the ability of higher CCT lighting conditions to accommodate the crew’s working requirements when operating the system, whether in the space station crew module or in the hygiene area environment. Furthermore, there are also ground-based studies that have concluded that environments with higher CCT conditions over a period of weeks do show alertness and revitalisation effects when participants are in a working environment for long periods of time, at least on self-report measures [19,67,68].

From a practical point of view, our findings can be extrapolated to demonstrate that CCT lighting levels may directly or indirectly affect comfort in other space station environments as well. According to different work tasks and living conditions, NASA has proposed LED lights with adjustable colour temperature and illuminance to optimise the living and working conditions of astronauts in an isolated and confined environment [69,70]. This can also support astronauts’ vision, regulate their neuroendocrine system, circadian rhythm and neurobehavior, and positively affect their sleep [71,72]. The use of 2700 K lighting in the sleeping area can help astronauts relieve fatigue, relax and fall asleep quickly. The use of 6500 K lighting in the core cabin with experimental cabinets can optimise work efficiency, increase alertness and reduce error rates [15,73]. This research has been applied to the current International Space Station and the Chinese Space Station. This further indicates that coloured lighting can impact not only the traditional user experience in the working area of a ground environment but also the comfort level of astronauts in isolated and confined space environments. In the space station environment, the matching of environmental lighting requirements to the visual space requirements within the area is an important factor in terms of comfort. Reasonable consideration of these factors in the design of space station areas can improve people’s sense of psychological identity and stimulate work efficiency. Otherwise, the environment may lead to discomfort, reduced work efficiency and even operational errors, which may, in severe cases, endanger the crew’s safety [74,75]. Therefore, further research in this direction may also consider the spatial orientation of astronauts, information acquisition and judgement, as well as psychological feelings, among other aspects. Thus, it can be concluded that the lighting design of a space station is related to the work efficiency and safety of the astronauts.

### Limitations and Future Research

The shortcomings of this study are as follows. The simulated environmental factors in the hygiene area of a space station during the experiment were limited. In this project, a high-fidelity simulation of the various systems in the hygiene area of a space station was constructed, but there is a large gap between the conditions in a real space laboratory and those in a simulated environment. In particular, when interacting with the equipment in microgravity, the crew may face more operational uncertainty and take longer to use the hygiene area. Future tests should be conducted on space stations and during parabolic flight simulations to simulate microgravity and air pressure states [75].

Regarding the design of the experiment, the process of selecting the sample and the criteria for the participants could be further improved. As astronauts or crew members in training usually undergo more rigorous mental, physical and behavioural training during the selection process, physical attributes such as endurance and agility will be somewhat different from those of ordinary participants. Therefore, in future experimental space station/laboratory environments, the test subjects should generally be chosen from special groups, such as trained astronauts, pilots and soldiers. These subjects are potential astronauts, which makes their physical and psychological characteristics closer to the standards of astronauts. Furthermore, although some studies have concluded that individual differences among participants in CCT illumination studies are greater than gender differences, it would still be possible to analyse gender and age group differences in the spacecraft environment in the future.

A number of studies have found different effects of different CCT conditions for morning and night. As human physiological and psychological functioning changes systematically over time, and as some previous findings suggest that daytime and nighttime effects are less consistent, it is important to explore potential differences in daytime and nighttime exposure [76].

The effects of CCT may also depend on the intensity of illumination. Whether the daytime effects of CCT on emotion, alertness and arousal levels are more pronounced at lower intensity levels, or whether higher CCT conditions and longer exposure times are necessary to induce beneficial effects on daytime physiological performance and subjective responses requires more research [77,78].

## 5. Conclusions

The results of this study contribute to the literature on the coloured lighting of space station environments in the following aspects. Firstly, we demonstrated that the CCT of lighting impacted the participants’ GSR, emotion, and subjective satisfaction in the simulated environment of a space station hygiene area. Secondly, we found that when the participants used the hygiene area simulator in the five CCT conditions tested, they had the best emotion and satisfaction in the 6300 K environment, which provides a valuable reference for improving the lighting in the space station hygiene area in the future. Furthermore, this study provides the manned spaceflight science community with a new potential paradigm for future lighting effects in manned spacecraft for different functional and operational areas based on the contribution of CCT lighting to physiological and subjective responses in simulations of operational processes in the hygiene area of the space station. We recommend further work to be done and suggest that a longitudinal design be used to replicate and extend our findings in order to study the impact of the CCT of lighting on a space station’s habitability with microgravity effects. The findings from this study provide valuable information about the use of coloured lighting in the hygiene area to improve the habitability design of future space station and spacecraft environments.

## Figures and Tables

**Table 1:** Duv and illuminance measured at desk level for each nominal CCT after the lighting animations had been set up.

	Nominal CCT (K)	Duv	Illuminance (Lux)
1	2700	0.0013	521
2	3000	−0.0010	558
3	3600	−0.0030	552
4	5000	0.0005	563
5	6300	0.0037	567

## Data Availability

The datasets used and/or analysed during the current study are available from the corresponding author upon reasonable request.
